# The Role of Light-Harvesting Complex II Organization in the Efficiency of Light-Dependent Reactions in the Photosynthetic Apparatus of *Pisum sativum* L.

**DOI:** 10.3390/plants14121846

**Published:** 2025-06-16

**Authors:** Georgi D. Rashkov, Martin A. Stefanov, Amarendra N. Misra, Emilia L. Apostolova

**Affiliations:** 1 Institute of Biophysics and Biomedical Engineering, Bulgarian Academy of Sciences, Acad. G. Bonchev Str., Bl. 21, 1113 Sofia, Bulgaria; megajorko@abv.bg (G.D.R.); martin_12.1989@abv.bg (M.A.S.); 2 Faculty of Sciences, Sri Sri University, Cuttack 754006, Odisha, India; misra.amarendra@gmail.com

**Keywords:** chlorophyll *a* fluorescence, LHCII, pea mutants, photosynthetic apparatus

## Abstract

In this study, the functions of the photosynthetic machinery were evaluated using chlorophyll *a* fluorescence technique (PAM and JIP test) in pea plants (*Pisum sativum* L. cv Borec) and its LHC II oligomerization variants (mutants *Costata 2*/*133* and *Coeruleovireus 2*/*16*). The oligomeric forms of LHCII increased in the following order: *Costata 2*/*133* < Borec wt < *Coeruleovireus 2*/*16*. Data revealed that the mutant with higher LHCII oligomerization (*Coeruleovireus 2*/*16*) at low light intensity (LL, 150 µmol photons/m^2^·s) exhibited the following: (i) decreased energy dissipation and increased electron transport efficiency; (ii) higher reaction center density; (iii) increased amounts of the open reaction centers (qp) and their excitation efficiency (Φexc); and (iv) influenced the reoxidation of Q_A^−^_, alleviating its interaction with plastoquinone. These effects enhanced photosynthetic performance related to PSII photochemistry (PI_ABS_) and overall photosynthetic efficiency (PItotal). High light intensity (HL, 500 µmol photons/m^2^·s) caused a reduction in open reaction centers (qp), excitation efficiency (Φexc), photochemical energy conversion of PSII (Φ_PSII_), maximum efficiency of PSII photochemistry in light (Fv′/Fm′), and linear electron transport via PSII, with more pronounced effects observed in membranes with a lower degree of LHCII oligomerization (*Costata 2*/*133*). This study provides novel experimental evidence for the pivotal role of the LHCII structural organization in determining the efficiency of light-dependent reactions of photosynthesis.

## 1. Introduction

Photosynthesis, which converts light energy into chemical energy, is highly sensitive to climate changes [1]. The primary reactions of photosynthesis in higher plants occur within complexes embedded in the thylakoid membranes that constitute the photosynthetic apparatus [2]. Four multisubunit membrane–protein complexes participate in these processes: photosystem I (PSI), photosystem II (PSII), cytochrome *b6f* (*cyt. b6f*), and ATP synthase [3]. These complexes ensure optimal activity of the light-dependent reactions of photosynthesis under varying light conditions [4]. It is known that the PSII and PSI complexes are located in distinct domains of the thylakoid membrane: PSII and its light-harvesting complex (LHCII) reside in appressed grana stacks, while PSI and its light-harvesting complex (LHCI) are in non-appressed stroma thylakoid membranes [5].

Peripheral antenna systems of both PSII and PSI are composed of light-harvesting chlorophyll a/b proteins [6], where the chlorophylls a function as a primary electron donor of reaction centers of both photosystems [6]. The light-harvesting complex of PSII is crucial for photosynthesis in plants. It captures and transfers light energy to both the photosystems and consists of proteins and pigments like chlorophylls and carotenoids, embedded in thylakoid membranes [7,8]. The LHCII consists of six Lhcb proteins and can exist in various forms, such as monomers, dimers, trimers, and aggregates, which switch in response to changes in light intensity [5,9,10]. The trimeric forms, which consist of proteins *Lhcb1*, *Lhcb2*, and *Lhcb3*, are particularly effective in capturing and transferring light energy in PSII [10]. Trimeric forms of LHCII are bound with the PSII core by *Lhcb4*, *Lhcb5*, and *Lhcb6* proteins. Phosphorylation influences LHCII’s organization within the thylakoid membrane, affecting energy distribution between photosystems. This dynamic organization is essential for optimizing light energy capture and ensuring efficient photosynthesis [5,10].

It is reported extensively that light intensity strongly affects the rate of the photosynthesis and the organization of LHCII, which is linked to changes in the levels of LHCII and the surface area of grana membranes [10,11]. In higher plants, low-light acclimation (shade plants) leads to an increase in the PSII antenna size and area of grana membranes [12,13], whereas sun plants exhibit a higher Chl *a*/*b* ratio, lower level of LHCII, and a lower degree of thylakoid stacking [1,14].

Chlorophyll *a* fluorescence is a fast, informative, and non-invasive method, widely used for the characterization of the photosynthesis of higher plants and gives information on the relationship between the structure and functions of the photosynthetic apparatus [15,16,17,18,19,20]. Pulse amplitude modulated (PAM) and chlorophyll a fluorescence induction are two measurement techniques widely used for characterization of the plant photosynthesis. A fast increase of the chlorophyll *a* fluorescence in dark-adapted leaves after applying light characterizes the primary photosynthetic processes. The energy trapping, electron transport, and dissipation of the energy in the antenna complexes depend on the structure of the photosynthetic apparatus [21].

Pigment mutants of higher plants are convenient models for studying the relationship between the structural organization of the complexes of the photosynthetic apparatus and their functions [22]. The study of pea chlorophyll mutant *Chlorotica* revealed that the reduction in the chlorophyll and carotenoid content corresponds with low photosynthetic activity [22]. It has been shown that the reduction of the chlorophyll content in mutants of maize and tomato leads to a decrease in the open PSII reaction centers (qp) and electron transport rate (ETR) [23,24]. The study of high pigment mutants in tomatoes (*Solanum lycopersicum* L.) demonstrates that they were characterized by higher net CO_2_ assimilation [25] and rate of photosynthesis [26]. Research on pea (*Pisum sativum* L.) mutants has shown that alterations in the amount and organization of LHCII modify the electric properties of thylakoid membranes, significantly impact energy distribution between both photosystems and affect the oxygen evolution [9,27].

The objects of the study in the present work are seedlings of *Pisum sativum* L. cv Borec (*wt*) and its mutants (*Costata 2*/*133* and *Coeruleovireus 2*/*16*). In previous investigations, the thylakoid membranes of these plants were characterized (Table 1). It has been shown that the oligomeric forms of LHCII increased in the following order: *Costata 2*/*133* < Borec wt < *Coeruleovireus 2*/*16*. The aim of this study is to assess how the degree of oligomerization of LHCII influences the light-dependent reactions of the photosynthetic apparatus. We used chlorophyll *a* fluorescence induction curves (OJIP curves) and pulse amplitude modulated (PAM) chlorophyll *a* fluorescence. The results give new information about the role of LHCII organization in the functional efficiency of the photosynthetic apparatus.

## 2. Results

### 2.1. Chlorophyll a Fluorescence Induction

**Figure 1:**
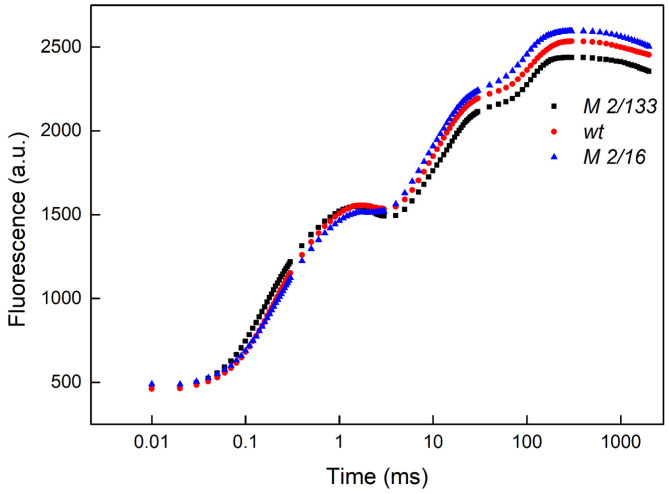
The representative OJIP chlorophyll *a* fluorescence curves in Borec wild type (wt) and its mutants *Costata 2*/*133* (M *2/133*) and *Coeruleovireus 2*/*16* (M *2/16*). (for measurement details see Section 4).

Chlorophyll *a* fluorescence induction gives information on the functions of the photosynthetic apparatus in studied pea plants. The fluorescence curve follows a polyphasic increase, denoted as OJIP [28,29], and gives information about PSII functions and the efficiency of the electron transport chain [30]. The curves of the chlorophyll *a* fluorescence induction for Borec wt and its mutants *Costata 2*/*133* and *Coeruleovireus 2*/*16* are given in Figure 1. Selected parameters of the chlorophyll *a* fluorescence induction (JIP test) were calculated, which provide a comprehensive overview of the performance and efficiency of the photosynthetic apparatus, encompassing aspects of light energy dissipation (DIo/RC), the stability of the OEC (Wk), and electron transport efficiency φE, ψE, φPo.

**Figure 2:**
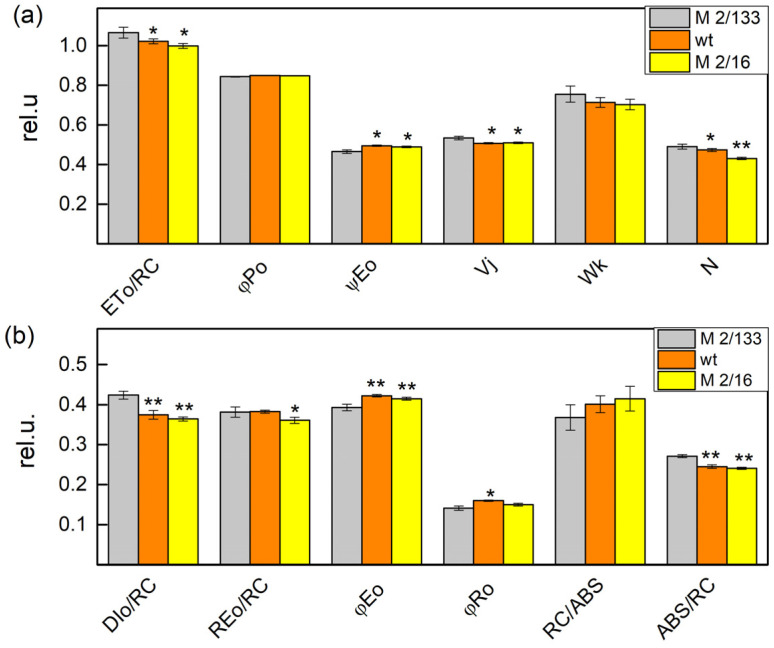
Selected JIP parameters in Borec wild type and its mutants *Costata 2*/*133* (M *2/133*) and *Coeruleovireus 2*/*16* (M *2/16*). The parameters include the following: ETo/RC—electron transport flux further Q_A^−^_ per reaction center; φPo—maximum quantum yield of primary photochemistry; ψEo—the efficiency of electron transport beyond Q_A_; Wk—the stability of the OEC; Vj—the fraction of closed reaction centers at the J-step in the chlorophyll *a* fluorescence curve; ABS/RC (×10^1^)—absorption per reaction center; RC/ABS—the ratio of active reaction centers per absorbed light; DIo/RC—light energy dissipation; φEo—the quantum yield of electron transport beyond Q_A_; φRo—the quantum yield of energy dissipation in the form of heat and fluorescence at the reaction center level; REo/RC—electron flux reducing end electron acceptors at the PSI acceptor side per reaction center; and N (×10^2^)—maximum turnover of Q_A_ reducing until Fm was reached. Significant differences between the plant with the lowest degree of LHCII oligomerization (*M 2/133*) and plants with a higher degree of oligomerization (wt and M *2/16*) were determined by Student’s *t* tests and are indicated by asterisks at *p* values less than 0.05 (*), 0.01 (**). (**a**) parameters: ETo/RC, φPo; ψEo, Vj, Wk, N; (**b**) parameters: Dio/RC, φEo, φRo, RC/ABS, ABS/RC.

**Figure 3:**
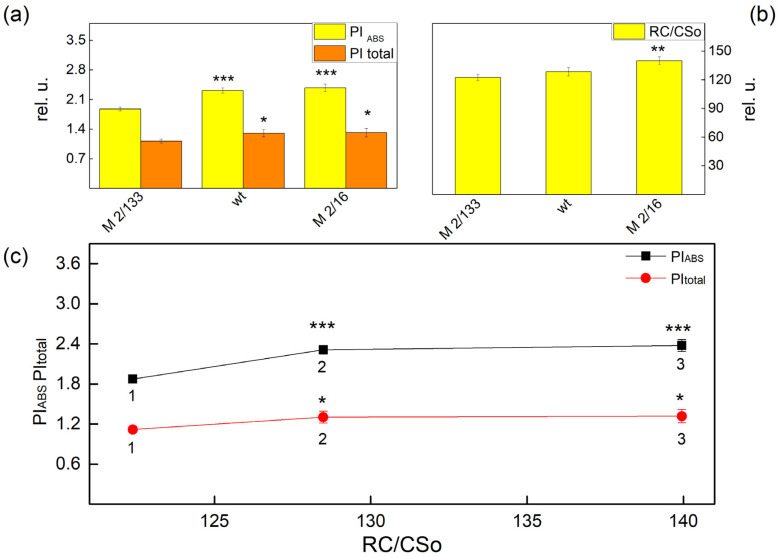
Performance indices PI_ABS_ and PItotal (**a**), the amount of active PSII per excited cross-section reaction center (RC/CSo), (**b**) in Borec wild type and its mutants *Costata 2*/*133* and *Coeruleovireus 2*/*16*. The relationship between the amount of active PSII per excited cross-section reaction center (RC/CSo) on the performance indices PI_ABS_ and PItotal. (**c**) 1, 2, 3 correspond with *Costata 2*/*133*, Borec wt, and *Coeruleovireus 2*/*16*, respectively. Significant differences between the plant with the lowest degree of LHCII oligomerization (*M* 2/133) and plants with a higher degree of oligomerization (wt and M *2/16*) were determined by Student’s *t* test and are indicated by asterisks at *p* values less than 0.05 (*), 0.01 (**), and 0.001 (***).

The comparison of the JIP parameters revealed differences between Borec wt and its mutants (Figure 2 and Figure 3). The data showed that the light absorption per reaction center (ABS/RC) or functional PSII antenna size, was slightly smaller (by about 10%) in *wt* and *mutant 2*/*16* than in the *mutant 2*/*133*, which reflects a bigger PSII antenna size in *mutant 2*/*133* than both *wt* and *mutant 2*/*16* (Figure 2). The plants with bigger oligomerization of LHCII (*wt* and *mutant 2*/*16*) have higher values of the parameter RC/ABS, φEo, φRo, and ψEo i.e., a higher number of the active reaction center per PSII antenna chlorophyll and better movement of the electron into electron transport chain beyond Q_A^−^_ (Figure 2). Dissipated energy (DIo/RC) decreased in membranes of *wt* (by 11%) and *mutant 2*/*16* (by 14%) in comparison to the *mutant 2*/*133*. Experimental results also revealed a decrease in ABS/RC, Wk, N, and Vj parameters in *wt* and *mutant 2*/*16* in comparison to *mutant 2*/*133* (Figure 2). The parameter φP was similar in all studied plants, i.e., the maximum quantum yield of primary photochemistry was not affected by the degree of the LHCII oligomerization (Figure 2).

Experimental results also revealed that the changes in the functional PSII antenna size influenced the performance indices (PI_ABS_ and PItotal) (Figure 2a). The increase of the ABS/RC corresponded with a decrease of both indices, i.e., ABS/RC increased while PI_ABS_ and PItotal decreased in the plants with a smaller degree of oligomerization (Figure 3a). At the same time the density of the reaction centers (RC/CSo, Q_A_-reducing reaction centers) was also bigger in the *wt* and *mutant 2*/*16* (Figure 3b). The correlation between the PI_ABS_ and PItotal and the amount of active PSII per excited cross-section reaction center (RC/CSo) are shown in Figure 3c.

**Figure 4:**
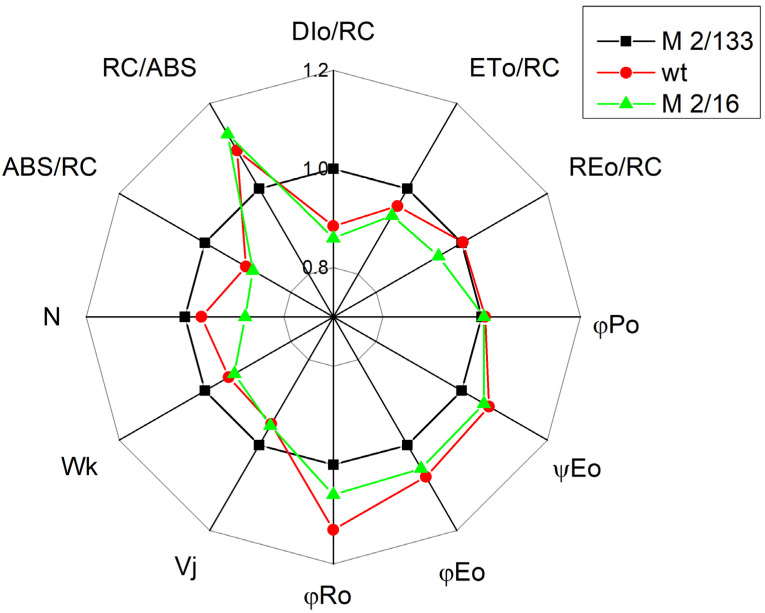
Radar plot representing various photosynthetic parameters in Borec wild type and its mutants *Costata 2*/*133* and *Coeruleovireus 2*/*16*. The parameters are the same as in Figure 2. The parameters are normalized to the plant with the lowest degree of LHCII oligomerization (*Costata 2*/*133*).

A comparison of the changes in the studied parameters in the plant with the lowest degree of oligomerization (*mutant 2*/*133*) with those in the plants with a higher degree of oligomerization (*wt* and *mutant 2*/*16*) is presented in Figure 4.

Significant differences between studied plants were found in the performance indices (PI_ABS_ and PItotal) (Figure 3a). The parameter PI_ABS_ was higher in *wt* (by 24%) and *mutant 2*/*16* (by 27%) than in *mutant 2*/*133*. The comparison of the PItotal of studied plants revealed that the values of this parameter were higher by 22% in the plants with a bigger oligomerization of LHII (*wt* and *mutant 2*/*16*) than its value in the *mutant 2*/*133*. The index PI_ABS_ is determined by the following: the number of active reaction centers per PSII antenna chlorophyll, γRC/(1 − γRC), the partial performance of primary photochemistry φPo/(1 − φPo), and the performance of thermal reactions of the intersystem electron carries ψ(Eo)/(1 − ψEo) [31,32,33]. Higher values of PIABS in *wt* and *mutant 2*/*16* were a result of high values of the component, characterized by non-light-dependent reaction [ψEo/(1 − ψ(Eo)] and the number of active reaction centers per PSII antenna chlorophyll [γRC/(1 − γRC)] (Table 2). These parameters determined the higher values of PItotal in *wt* and *mutant 2*/*16* than the values of this parameter in *mutant 2*/*133*, because the parameter [δREo/(1 – δREo)] has similar values in Borec wt and its mutants i.e., differences in the efficiency in the electron transfer from Q_B_ to PSI acceptors were not registered in studied plants.

### 2.2. PAM Chlorophyll a Fluorescence

**Figure 5:**
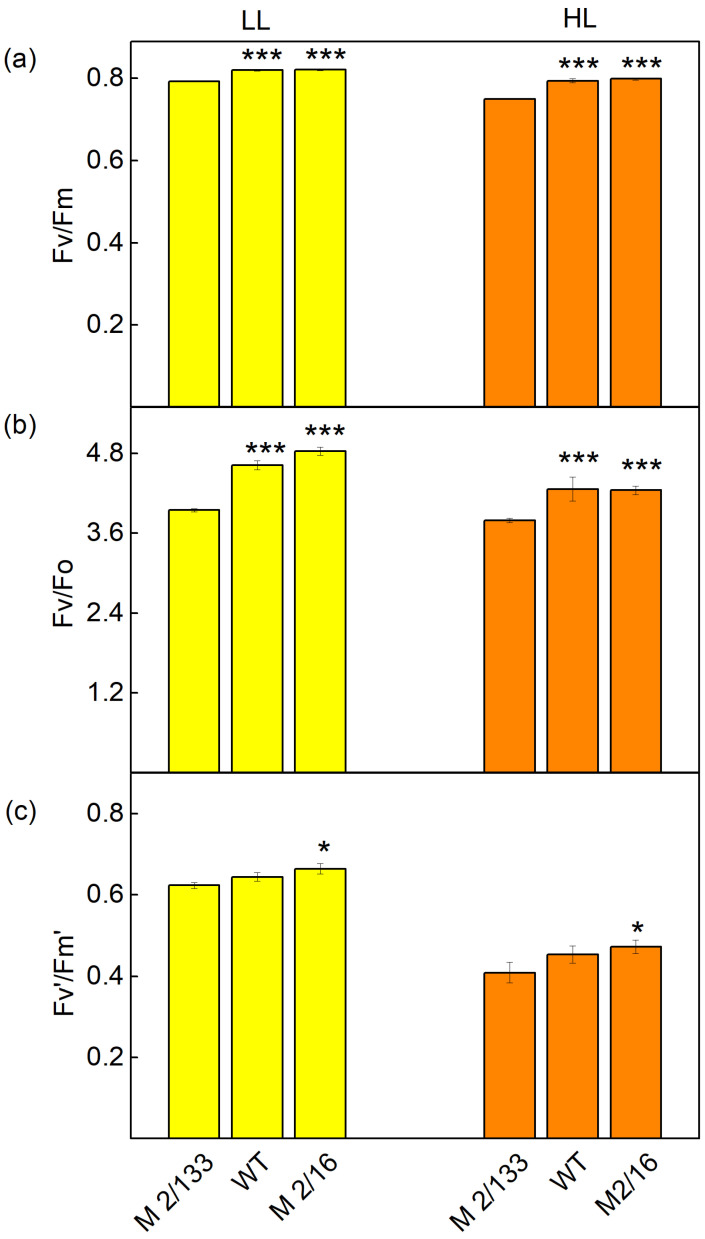
The parameters of PAM chlorophyll *a* fluorescence in Borec wild type (*wt*) and its mutants *Costata 2*/*133* (M *2/133*) and *Coeruleovireus 2*/*16* (M 2/16) at low light (LL, 150 μmol photons/m^2^·s actinic light) and high light (HL, 500 μmol photons/m^2^·s actinic light: (**a**) the maximal quantum yield) in dark-adapted state (Fv/Fm); (**b**) the intensity of chlorophyll *a* fluorescence caused by photochemical processes to intensity of the chlorophyll *a* fluorescence not excitonically bound to the reaction centers of PSII (Fv/Fo); (**c**) the effective quantum yield of PSII photochemistry (Fv′/Fm′). Significant differences between the plant with the lowest degree of LHCII oligomerization (*M 2/133*) and plants with a higher degree of oligomerization (wt and M *2/16*) were determined by Student’s *t* tests and are indicated by asterisks at *p* values less than 0.05 (*) and 0.001 (***).

**Figure 6:**
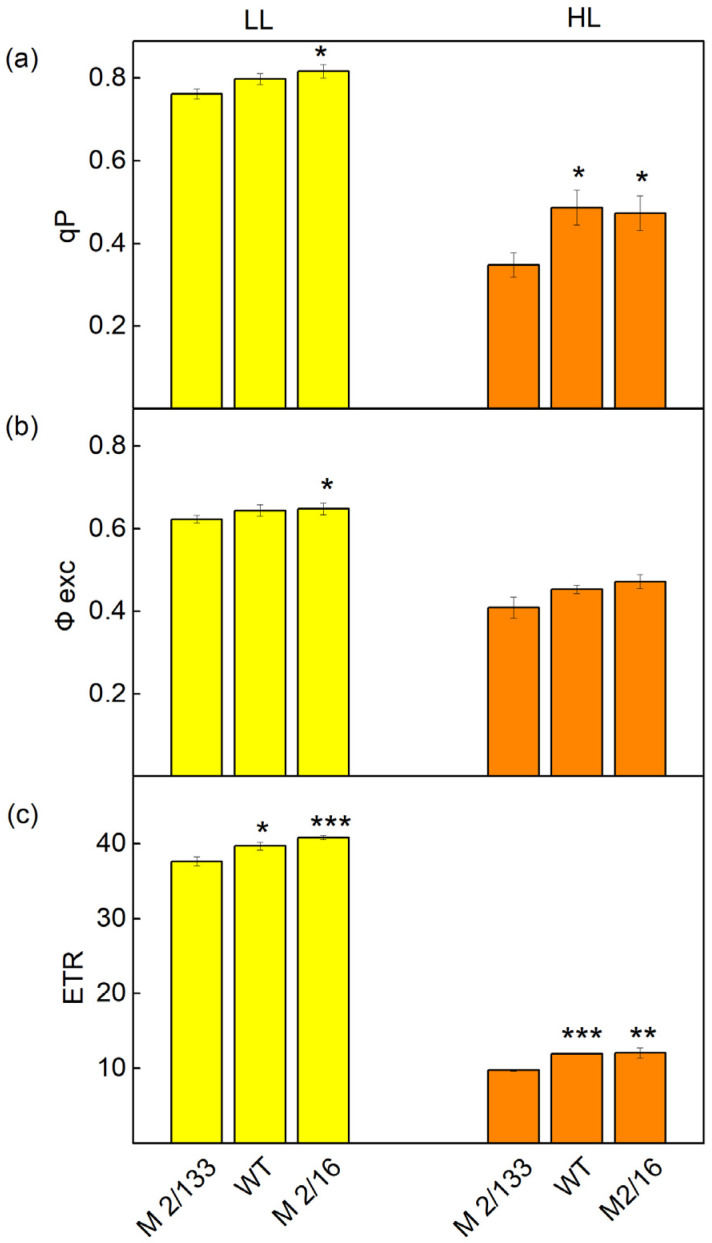
The parameters of PAM chlorophyll *a* fluorescence in Borec wild type and its mutants *Costata 2*/*133* and *Coeruleovireus 2*/*16* at low light (LL, 150 μmol photons/m^2^·s actinic light) and high light (HL, 500 μmol photons/m^2^·s actinic light: (**a**) the photochemical quenching (qp); (**b**) excitation efficiency of open PSII center (Φexc); (**c**) the linear electron transport (ETR). Significant differences between the plant with the lowest degree of LHCII oligomerization (*M 2/133*) and plants with a higher degree of LHCII oligomerization (wt and M 2/16) were determined by Student’s *t* test and are indicated by asterisks at *p* values less than 0.05 (*), 0.01 (**), and 0.001 (***).

**Figure 7:**
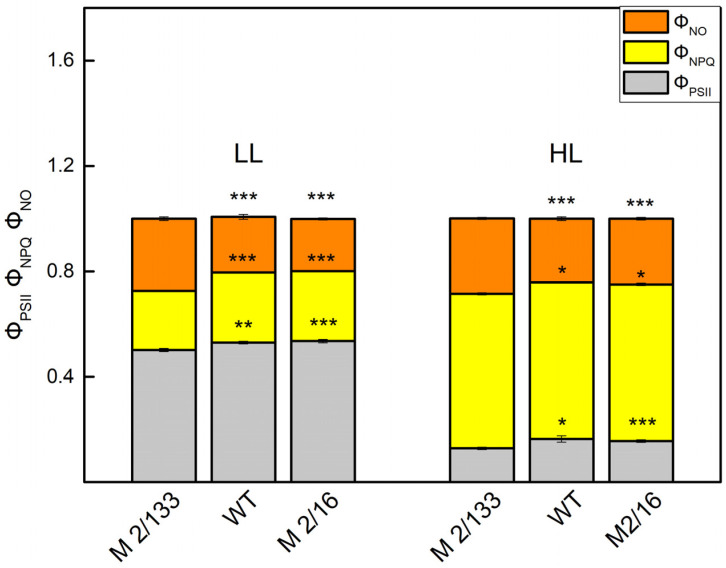
The effective quantum yield of photochemical energy conversion of PSII (Φ_PSII_), and the regulated (Φ_NPQ_) and non-regulated (Φ_NO_) energy loss in Borec wild type (wt) and its mutants *Costata 2*/*133* (M *2/133*) and *Coeruleovireus 2*/*16* (M 2/16) at low light (LL, 150 μmol photons/m^2^·s actinic light) and high light (HL, 500 μmol photons/m^2^·s actinic light). Significant differences between the plant with the lowest degree of LHCII oligomerization (M *2/133*) and plants with a higher degree of oligomerization (wt and M 2/16) were determined by Student’s *t* test and are indicated by asterisks at *p* values less than 0.05 (*), 0.01 (**), and 0.001 (***).

The PAM chlorophyll *a* fluorescence signals revealed differences in studied parameters in pea plants (Borec wild type and its mutants *Costata 2*/*133* and *Coeruleovireus 2*/*16*) (Figure 5, Figure 6 and Figure 7). The data also showed an influence of actinic light during the following measurements: a low light (LL, 150 μmol photons/m^2^·s actinic light) or a high light (HL, 500 μmol photons/m^2^·s actinic light). The maximum efficiency of PSII photochemistry (Fv/Fm) was higher by 3% at LL and 6% at HL in *mutant 2*/*16* and wild type compared to *mutant 2*/*133* (Figure 5). The ratio of the intensity of chlorophyll *a* fluorescence caused by photochemical processes to the intensity of the chlorophyll *a* fluorescence not excitonically bound to the reaction centers of PSII (Fv/Fo) was bigger in *wt* and *mutant 2*/*16* (LL by 17–22%, HL by 33%) in comparison to the *mutant 2*/*133*.

Differences in the organization of the photosynthetic apparatus also affect the following: the effective quantum yield of PSII photochemistry (Fv′/Fm′), photochemical quenching (qp), excitation efficiency of open PSII center (Φexc), and linear electron transport rate (ETR). At low light (LL), a slight increase in Fv′/Fm′ (by 3–6%), qp (by 5–7%), and Φexc (by 4%) were registered in *wt* and *mutant 2*/*16* in comparison to the *mutant 2*/*133* (Figure 5 and Figure 6). The differences between studied plants were bigger at HL. The photochemical quenching (qp) was higher from 36% to 40% and ETR from 20% to 21% in *wt* and *mutant 2*/*16* than the values of these parameters in *mutant 2*/*133*. The parameters Φexc and Fv′/Fm′ were bigger by 11–13% in *wt* and *mutant 2*/*16* than in *mutant 2*/*133* (Figure 6).

The energy absorbed by PSII is the sum of yields of photochemical energy conversion in PSII (ΦPSII), regulated (Φ_NPQ_), and non-regulated energy losses (Φ_NO_) [34]. At LL, the parameter Φ_PSII_ was higher (5–8%) in plants with higher degree of oligomerization of LHCII (*wt* and *mutant 2*/*16*) than in the *mutant 2*/*133* (Figure 7). At the same time, Φ_NO_ was smaller (by about 25%), while Φ_NPQ_ was bigger (by about 20%) in leaves of *wt* and *mutant 2*/*16* in comparison to those in the *mutant 2*/*133* (Figure 7).

Non-photochemical quenching of chlorophyll *a* fluorescence (NPQ) is essential for plant photoprotection [35]. The components that NPQ involves are as follows: energy-dependent quenching (qE), state transition quenching (qT), and photoinhibitory quenching (qI). The values of the component qE were similar in *wt* and studied mutants except for *mutant 2*/*16* at HL (Table 3). The state transition quenching (qT) is about 9–10 times higher at LL and about 2 times at HL in wt and *mutant 2*/*16* compared to *mutant 2*/*133*. Data also revealed a higher value of the qI in plants, with a higher degree of LHCII oligomerization (*wt* and *mutant 2*/*16*), by 41–45% at LL and by 12–14% at HL (Table 3).

We estimated the influence of the LHCII organization on the Q_A^−^_ reoxidation by measuring the dark reduction of the chlorophyll *a* fluorescence after single saturated light pulse. Two components of the fluorescence signal characterized two pathways of Q_A^−^_ reoxidation [36,37]. Component A_1_, with rate constant k_1_, characterizes the interaction of Q_A_ with a plastoquinone, while component A_2_, with rate constant k_2_, characterizes an interaction of Q_A_ with the oxygen-evolving complex. The constant k_1_ data revealed that it did not differ in *wt* and two mutants, while the constant k_2_ was higher in *mutant 2*/*133* than in *wt* and *mutant 2*/*16* (Table 4). At the same time, the ratio of two components (A_1_/A_2_) was smaller in *mutant 2*/*133* in comparison to the *wt* and *mutant 2*/*16*.

### 2.3. Principal Component Analysis (PCA)

**Figure 8:**
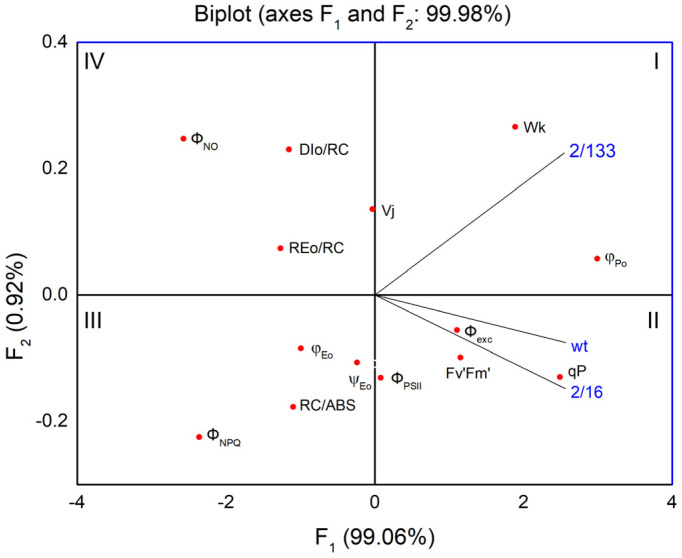
Principal component analysis (PCA) shows variation among Borec wild type and its mutants *Costata 2*/*133* and *Coeruleovireus 2*/*16* (blue lines) in relation to selected parameters of chlorophyll *a* fluorescence (PAM and JIP test) shown as red dots.

The first two components (Figure 8 and Table S1) account for 99.98% of the variability in the data. The pea mutant with the lowest oligomerization of LHCII complexes (*mutant 2*/*133*), positioned in the first quadrant, exhibits a negative correlation/relationship with parameters related to the number of active reaction centers per PSII antenna chlorophyll (RC/ABS), the quantum yield of electron transport (φEo), and the regulated energy losses from PSII (Φ_NPQ_), which are located in the third quadrant. Simultaneously, a more distinct positive correlation is observed in the *mutant 2*/*133* for processes associated with non-photochemical quenching per reaction center unit (DIo/RC), nonregulated energy losses of PSII (Φ_NO_), and the stability of the OEC (Wk), all of which are located on the upper side of the F1 axis. In contrast, the *mutant 2*/*16* and *wt*, located in the second quadrant and characterized by higher oligomerization of the LHII than *mutant 2*/*133*, show significantly improved photochemical activity, as indicated by the parameters in the same quadrant (Fv′/Fm′, Φexc, qp, Φ_PSII_).

## 3. Discussion

The regulatory role of LHCII in photosynthesis is well known [38,39,40]. The thylakoid membranes of studied pea plants are characterized by varying amounts and degrees of oligomerization of LHCII [27]. The ratio of oligomeric to monomeric forms of LHCII increases in the following order *mutant 2*/*133* < *wt* < *mutant 2*/*16* (Table 1). In this study, the functions of the photosynthetic apparatus are characterized in detail. A deeper understanding of the role of LHCII in primary photochemistry may help to understand the mechanisms of photosynthetic efficiency.

Variation of the chlorophyll content influences the absorbed light energy per active center (ABS/RC) (Figure 3 and Figure 4). The parameter ABS/RC was smaller in the membranes with a higher ratio of LHCIIo/LHCIIm, which reflects a smaller apparent antenna size in the membrane with a bigger degree of LHCII oligomerization. It has been shown that the ratio LHCII/PSII is higher in the plant (*mutant 2*/*133*) than *wt* and *mutant 2*/*16* (Table 1), which suggests the synthesis of additional LHCII proteins [41]. The authors suggest that these additional synthesis proteins belong to the population of the LHCII molecules non-attached to PSII. This observation could explain the smaller apparent antenna size in the membrane with a bigger degree of LHCII oligomerization. At the same time, an increase in the number of active reaction centers per PSII antenna chlorophyll (RC/ABS) in the membrane with a bigger amount of the LHCIIo was registered. The data also showed a slight increase in the density of the reaction center (RC/CSo) in thylakoid membranes with the biggest amount of LHCIIo (*mutant 2*/*16*).

The experimental results revealed a variation in the parameters Wk and Vj, which could result from differences in the donor and acceptor side of PSII, respectively. The differences in parameter Wk in studied plants revealed an influence on the function of the OEC (Figure 2 and Figure 4) and correspond with changes in the kinetic parameters of the oxygen evolution, as a result of the modification of the Mn clusters and an influence on the oxygen-evolving reactions [27]. The influence of the donor side of the PSII complex could be connected by changes in the conformation of the OEC and/or of the proteins surrounding the complex and from variations of the surface electric parameters of the membrane [9]. The differences in parameter Fv/Fo in *wt* and mutants, which connected with the efficiency of the OEC [42,43,44] also revealed some differences in the donor side of PSII (Figure 2 and Figure 4).

Changes in the LHCII oligomerization are associated with modification not only on the donor side but also on the acceptor side of the PSII complex. The parameter Vj characterized the fraction of Q_A_ in the reduced state [45] was also influenced (Figure 2 and Figure 4). The current study showed that LHCII oligomerization influences the pathways of Q_A^−^_ reoxidation (Table 4). The constant characterizing Q_A^−^_ reoxidation through plastoquinone (k_1_) was similar in Borec wt and its mutants, but the constant k_2_, characterizing the interaction with OEC decreased in the membranes with a higher degree of LHCII oligomerization. At the same time, the ratio A_1_/A_2_ increased in *wt* and *mutant 2*/*16*, i.e., the electron flow from Q_A_ to plastoquinone is facilitated. The higher value of the Vj and smaller of the ψEo in *mutant 2*/*133* compared with *wt* and *mutant 2*/*16* (Figure 2) suggests decreased electron transfer beyond Q_A_ in this mutant, representing smaller electron movement through the electron transport chain.

At the same time, plants with a smaller amount of LHCIIo are characterized by increased dissipated energy flux per reaction center (DIo/RC) (Figure 2 and Figure 4). In addition, the data also revealed that the regulated energy losses (Φ_NPQ_) were bigger than the non-regulated energy losses (Φ_NO_) in all studied plants at HL actinic light (Figure 7). At the same time, Φ_NO_ was bigger in *mutant 2*/*133* in comparison to the plants with bigger LHCII oligomerization (wt and *mutant 2*/*16*). One of the reasons for high Φ_NO_ at a smaller degree of oligomerization of LHCII could be due to changes in the organization of the OEC in comparison to the plants with higher oligomerization of LHCII. The differences in the organization of the OEC are connected with variations in the ratio of active PSIIα to PSIIβ centers as well as an influence on the So-S_1_ state distribution in darkness [9]. Furthermore, it was found that the modification of OEC is associated with high rates of non-radiative charge recombination between P680+ and Q_A_ [46]. The non-regulated energy losses (Φ_NO_) reflect energy quenching processes occurring within the PSII reaction center with Q_A_ in a reduced state [47]. Moreover, the reduction of Q_A_ has been suggested to be a major requirement for an efficient PSII reaction center-derived quenching [48]. The experimental data revealed that Q_A_ is more reduced in the plant with a smaller degree of the LHCII oligomerization (*mutant 2*/*133)* (Figure 6) and Φ_NO_ values are significantly higher (Figure 7).

The components of non-photochemical quenching give more information about the dissipative mechanism in the thylakoid membrane (qE, qT, and qI) (Table 3). The data revealed that the parameter qT was higher in *wt* and *mutant 2*/*16* in comparison to *mutant 2*/*133*. It is well known that state transition quenching (qT) is a process that redistributes excitation energy between two photosystems and it is very important for the protection of the photosynthetic apparatus [49,50]. The component qE was similar in all studied plants, but strongly increased at HL in all studied plants, as the increase is more pronounced in *mutant 2*/*16* (Table 3), which supports a previous statement that qE protects PSII against short-term high light and fluctuations in light intensities [51]. It could be suggested/concluded that plants with higher LHCII oligomerization have better protection against high light.

The different organization of the LHCII influenced the amount of the open reaction center (qp) and their excitation efficiency (Φexc), as well as the electron transport rate (ETR) (Figure 6). These parameters had smaller values in the photosynthetic apparatus with a decreased amount of LHCIIo, i.e., in *mutant 2*/*133* characterized by decreased pigment content and a smaller degree of oligomerization of LHCII. It could be concluded that differences in the organization of the LHCII-PSII complex, which corresponds with variation in the OEC and its functions [9], as well as an influence on the acceptor side (Vj) determined the differences in qp and (Φexc) in studied plants. The reduction of the parameters qp and ETR was also shown in maize mutants with reduced chlorophyll content [23]. A decrease of the parameter qp was also registered at the yellow left mutant of tomato (YLM) [24]. In addition, this study showed that the reduction of open centers in studied plants with decreased chlorophyll content and a small amount of LHCIIo was associated with a decrease in their efficiency (Figure 5). Moreover, at HL, the parameters qp, Φexc, and ETR decreased but these parameters were higher in the membrane with a bigger degree of oligomerization of LHCII (*wt* and *mutant 2*/*16*) (Figure 6). The different responses to changes in abiotic factors were observed in the yellow left mutant of tomatoes (YLM). The study of this mutant revealed higher sensitivity to low temperatures [24].

Performance indices reveal the photosynthetic performance related to PSII photochemistry (PI_ABS_) and the overall photosynthetic efficiency (PItotal) [21]. Data showed that both indices were bigger in *wt* and *mutant 2*/*16*, i.e., in the membranes with a bigger density of the reaction centers (RC/CS) and LHCII oligomerization (Figure 2). The differences of both performance indices were determined from the performance of the thermal reactions of the intersystem electron carries ψ(Eo)/(1 − ψEo) and the number of active reaction centers per PSII antenna chlorophyll, γRC/(1 − γRC) (Table 2), i.e., at the same time, differences in the efficiency in the electron transfer from Q_B_ to PSI acceptors were not registered in studied plants. Data revealed that the better efficiency of the function of the light-dependent reactions of the photosynthetic apparatus is connected with better photosynthetic performance of the PSII photochemistry.

## 4. Materials and Methods

### 4.1. Plant Material

Plants from wild type peas (*Pisum sativum* L. Borec) and two pea mutants (*Costata 2*/*133* and *Coeruleovireus 2*/*16*) were grown in pots containing ½ Hoagland solution (pH 6.5). The characteristics of the plants from the previous study are given in Table 1. The composition of the nutrient medium is as follows: 2.5 mM KNO_3_, 2.5 mM Ca(NO_3_)_2_, 1 mM MgSO_4_, 0.5 mM NH_4_NO_3_, 0.5 mM K_2_HPO_4_, 23 µM H_3_BO_3_, 4.5 µM MnCl_2_, 0.4 µM ZnSO_4_, 0.2 µM CuSO_4_, 0.25 µM Na_2_MoO_4_, and 20 µM Fe-EDTA (pH 6.0). The solutions were changed every 3 days. Each pot contained 10 seedlings. Plants were maintained under growth under controlled conditions of 12 h light/dark photoperiod; 19 °C/23 °C night/day is the temperature; 150 µmol photons/m^2^·s is the light intensity; and 65% humidity. After two weeks (14 days) of growing, the leaves of the seedlings were collected, measured, and analyzed. Fully developed leaves were used for analysis. Three independent experiments were conducted. Four plants per experiment were made for each explored plant type.

### 4.2. Fast Chlorophyll a Fluorescence Kinetics

Fast chlorophyll fluorescence induction kinetics were measured using the Handy PEA fluorimeter (Hansatech Instruments, King’s Lynn, UK). Prior to measurement, samples were dark-adapted for 20 min using specialized leaf clips to allow full relaxation of photosynthetic components. The fluorimeter’s high-intensity saturating light pulse (up to 3000 µmol photon/m^2^·s) enabled precise quantification of fluorescence dynamics, capturing rapid changes in photosystem activity. Illumination was provided by an array of three light-emitting diodes. The applied light pulse lasted for 1 s, ensuring optimal excitation conditions. The recorded fluorescence data were subsequently utilized for the computation of JIP-test parameters, enabling a detailed assessment of photosynthetic performance. Key parameters analyzed included the following [28]:

Mo = TRo/RC − ETo/RC is the initial slope;

ABS/RC = Mo·(1/Vj)·(1/Φ_Po_) is the absorption per reaction center;

RC/CSo = (ABS/CS)/(ABS/RC) the number of active PSII reaction centers per excited cross-section;

RC/ABS = 1/ABS/RC is the ratio of active reaction centers per absorbed light;

ψEo = 1 − V_j_ is the efficiency that a trapped exciton can move an electron further than Q_A^−^_ into the electron transport chain;

DIo/RC = (ABS/RC) − (TR_0_/RC) is the light energy dissipation;

Wk − F_300 μs_/F_2 ms_ represents the ratio between the specific rises in the fluorescence curve during the J phase and the K phase;

φPo = Fv/Fm is the quantum yield of primary photochemistry; the maximum quantum yield of primary photochemistry, representing the efficiency of energy conversion in PS II;

Vj = (F_2ms_ − Fo)/(Fm − Fo) indicates the fraction of closed reaction centers at the J-step in the chlorophyll *a* fluorescence curve;

φRo = Fv/Fm (1 − V_j_) is the quantum yield of energy dissipation in the form of heat and fluorescence at the reaction center level;

φEo = Fv/Fm (1 − V_I_)—the quantum yield of electron transport beyond Q_A_ (the primary quinone electron acceptor);

REo/RC = M_0_ (1/V_J_) (1 − V_I_)—the rate of electron transport per reaction center, indicating the efficiency of electron flow through the photosynthetic system;

ETo/RC = M_0_ (1/V_J_) (1 − V_J_)—the electron transport rate per reaction center, reflecting the overall electron transport activity;

N = Sm·M_0_·(1/V_J_) turn-over number Q_A^−^_ the relative size of the plastoquinone pool, which is involved in electron transport within the photosynthetic apparatus.

The performance indices PI_ABS_ (energy conversion from exciton to the reduction in intersystem electron acceptors) and PItotal (energy conversion from exciton to the reduction in PSI end acceptors) were also determined. The equations for their calculation are as follows [52]:

PI_ABS_ = RC/ABS × φPo/(1 − φPo) × ψEo/(1 − ψEo);

PItotal = PIABS × δREo/(1 − δREo).

Measurements were processed using the PEA+ software 1.13 for detailed OJIP analysis.

### 4.3. PAM Chlorophyll a Fluorescence Measurements

PAM chlorophyll fluorescence measurements at ambient temperature were carried out on dark-adapted leaf discs (1 cm in diameter) using a PAM fluorometer (model 101/103, Heinz Walz GmbH, Effeltrich, Germany) as in Stefanov et al. [53]. The minimum fluorescence level (F_o_) was recorded after 20 min of dark adaptation by applying a weakly modulated measuring light (0.02 µmol photons/m^2^·s). The maximum fluorescence levels in the dark-adapted (Fm) and light-adapted (Fm′) states were determined using a 0.8 s saturating pulse at 3000 µmol photons/m^2^·s. Two actinic light (AL) intensities were applied: 150 µmol photons/m^2^·s (low light, LL) and 500 µmol photons/m^2^·s (high light, HL). The steady-state fluorescence level (F_0_′) was assessed 5–6 min after the onset of actinic illumination. Selected chlorophyll *a* fluorescence parameters were calculated as follows: the maximum quantum efficiency of PSII in the dark-adapted state (F_v_/F_m_ = (F_m_ − F_0_)/F_m_), the ratio of quantum yields of photochemical and concurrent non-photochemical processes in PSII, Fv/Fo = (Fm − Fo)/Fo the effective quantum yield of energy conversion in PSII (Φ_PS_II = (Fm′ − Fs)/Fm′), the photochemical quenching coefficient (qp = (F_m_′ − Fs)/F_v_′), the linear electron transport rate, ETR = Φ_PSII_ × 150 × 0.5 × 0.84; the non-regulated (Φ_NO_ = Fs/Fm) and regulated (Φ_NPQ_ = Fs/Fm′ − Fs/Fm) energy loss in PSII; the effective quantum yield of PSII photochemistry, Fv′/Fm′ = (Fm′ − Fo′)/Fm′ [54,55]. The excitation efficiency of open PSII reaction centers (Φexc = Φ_PSII_/qp) was calculated using the Genty et al. [56] approach.

The kinetic constants (k_1_ and k_2_) and the amplitude ratio (A_1_/A_2_) of the decay of variable chlorophyll *a* fluorescence relaxation after excitation by a saturating light pulse in dark-adapted leaves were also assessed [21,36,37,57].

The components of non-photochemical quenching: qE—the energy-dependent quenching component, qT—the state-transition quenching component, and qI—the photoinhibitory quenching component were determined as described in [58].

### 4.4. Principal Component Analysis (PCA)

Principal Component Analysis (PCA), a complex statistical technique, was applied to diminish a broad set of measured parameters into the most essential ones [59]. PCA was further employed to assess the influence of different LHCII oligomerization features of *Pisum sativum* L. Borec (wt) and two pea mutants (*Costata 2*/*133* and *Coeruleovireus 2*/*16*) on JIP and PAM-derived fluorescence parameters. To determine the variability in response to structural changes/modifications in the LHCII complex, a clustering algorithm was utilized [60]. Multivariate statistical analysis using PCA was carried out, and a graphical illustration was created using Origin 9—software for data processing (OriginLab Corporation, Northampton, MA, USA). Meanwhile, the assumption of homoscedasticity is satisfied by the obtained data. Statistical significance between means was assessed using Student’s *t*-test. Differences were considered significant for *p* values < 0.05 (*), *p* < 0.01 (**), and *p* < 0.001 (***).

## 5. Conclusions

**Figure 9:**
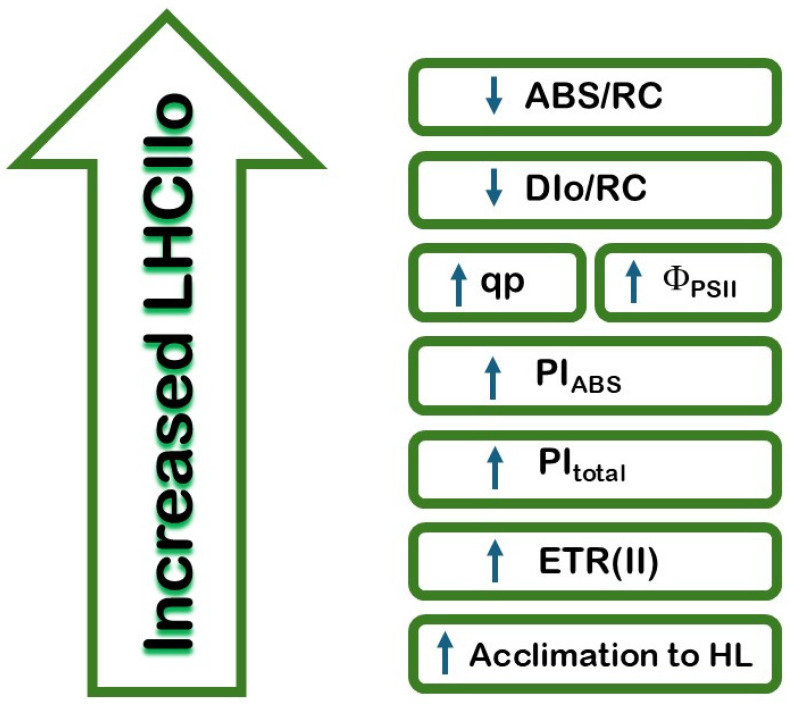
Diagram of the main effects of LHCII oligomerization on the efficiency of the photosynthetic apparatus.

This study highlights the critical regulatory role of LHCII oligomerization in photosynthetic efficiency. The influence of the LHCII oligomerization on the light reaction of photosynthesis is summarized in Figure 9. The current study, for the first time, revealed that variations in LHCII organization significantly influence parameters such as energy absorption and electron transport efficiency. Increased LHCII oligomerization correlates with higher reaction center density and improved photosynthetic performance indices (PI_ABS_ and PItotal), which could be a result of an influence on the reoxidation of the Q_A^−^_. Conversely, lower LHCII oligomerization is linked to increased energy dissipation and reduced electron transport efficiency. These findings suggest that LHCII’s structural organization plays a pivotal role in optimizing photosynthetic function under different environmental conditions. Insights into these mechanisms deepen our understanding of photosynthetic efficiency and its adaptation to abiotic factors. The conclusions regarding the role of LHCII oligomerization in the efficiency of the photosynthetic apparatus could be used to assess the possibility of evaluating the stability of new plant varieties in various environmental conditions.

## Figures and Tables

**Table 1:** Characteristics of the chlorophyll content and ratio of chlorophyll protein complexes in thylakoid membranes isolated from wild type pea (*Pisum sativum* L. cv. Borec) and its mutants (*Costata* 2/133 and *Coeruleovireus 2/16*). The determination was made by nondenaturating SDS–PAGE. LHCIIo and LHCIIm are the oligomeric and monomeric forms of the major LHCII, respectively [9,27].

Variant	Chl a + b (µg/g FW)	Chl a/b	LHCIIo/LHCIIm	LHCII/PSII	PSI/PSII
*Costata 2*/*133*	1840	2.54	3.34	3.70	1.27
Borec *wt*	2111	2.41	4.57	3.37	1.38
*Coeruleovireus 2* */16*	2680	2.23	6.62	3.21	1.37

**Table 2:** Components of the performance indices PI_ABS_ and PI_total_ in Borec *wild type* and its mutants *Costata 2*/*133* and *Coeruleovireus 2*/*16.* The parameters are in relative units. Significant differences between the plant with the lowest degree of LHCII oligomerization (*mutant 2*/*133*) and plants with a higher degree of oligomerization (*wt* and *mutant 2*/*16*) were determined by Student’s *t* tests and are indicated by asterisks at *p* values less than 0.05 (*).

	γRC/(1 − γRC)	φPo/(1 − φPo)	ψEo/(1 − ψ(Eo))	δREo/(1 − δREo)
*mutant 2*/*133*	0.372 ± 0.005	5.384 ± 0.079	0.893 ± 0.031	0.581 ± 0.035
*wt*	0.415 ± 0.006 *	5.536 ± 0.062	0.968 ± 0.014 *	0.590 ± 0.018
*mutant 2*/*16*	0.415 ± 0.004 *	5.549 ± 0.059	0.996 ± 0.018 *	0.561 ± 0.018

**Table 3:** Components of the non-photochemical quenching of chlorophyll *a* fluorescence in Borec wild type and its mutants *Costata 2*/*133* and *Coeruleovireus 2*/*16*. qE—energy-dependent quenching; qT—state transition quenching; and qI—photoinhibitory quenching. Significant differences between the plant with the lowest degree of LHCII oligomerization (*mutant 2*/*133*) and plants with a higher degree of oligomerization (wt and *mutant 2*/*16*) were determined by Student’s *t* test and are indicated by asterisks at *p* values less than 0.05 (*), 0.01 (**), and 0.001 (***).

	qE		qT		qI	
	LL	HL	LL	HL	LL	HL
*mutant 2*/*133*	0.62 ± 0.07	1.32 ± 0.07	0.010 ± 0.001	0.092 ± 0.021	0.094 ± 0.010	0.127 ± 0.002
*wt*	0.66 ± 0.06	1.34 ± 0.04	0.088 ± 0.009 ***	0.178 ± 0.011 **	0.137 ± 0.007 **	0.143 ± 0.019 *
*mutant 2*/*16*	0.65 ± 0.06	1.46 ± 0.03 *	0.097 ± 0.015 ***	0.182 ± 0.036 *	0.133 ± 0.028 **	0.141 ± 0.008 *

**Table 4:** Kinetic characteristics of the dark relaxation of chlorophyll fluorescence induced by a single saturating light pulse in Borec wild type and its mutants *Costata 2*/*133* (*mutant 2*/*133*) and *Coeruleovireus 2*/*16* (*mutant 2*/*16*): k_1_—constant of the fast component; k_2_—constant of the slow component; A_1_/A_2_—the ratio of the fast and slow components. Significant differences between the plant with the lowest degree of LHCII oligomerization (*mutants 2*/*133*) and plants with a higher degree of oligomerization (wt and *mutants 2*/*16*) were determined by Student’s *t* test and are indicated by asterisks at *p* values less than 0.05 (*) and 0.001 (***).

	k_1_ (s^−1^)	k_2_ (s^−1^)	A_1_/A_2_
*mutant 2*/*133*	1.733 ± 0.095	0.080 ± 0.004	4.231 ± 0.229
*wt*	1.596 ± 0.197	0.065 ± 0.003 ***	4.859 ± 0.221 *
*mutant 2*/*16*	1.609 ± 0.176	0.060 ± 0.004 ***	4.855 ± 0.252 *

## Data Availability

Data are contained within the article and Supplementary Materials.

## Supplementary Materials

The following supporting information can be downloaded at https://www.mdpi.com/article/10.3390/plants14121846/s1, Table S1. Contributions of variables (loadings) for the principal component analysis model shown in Figure 7.
