# Supplementary material for: The Role of Light-Harvesting Complex II Organization in the Efficiency of Light-Dependent Reactions in the Photosynthetic Apparatus of Pisum sativum L
Source: Plants (Basel). 2025 Jun 16;14(12):1846. doi: 10.3390/plants14121846 (PMC12196630; doi:10.3390/plants14121846)
Supplement: Supplementary file 1 [file plants-14-01846-s001.zip › plants-3640102-supplementary.pdf]

Role of Light Harvesting Complex II Organization for the Efficiency of the Photosynthetic Apparatus

Georgi D. Rashkov[1], Martin A, Stefanov[1], Amarendra N. Misra[2] and Emilia L. Apostolova[1*]

Table S1. Contributions of variables for the principal component analysis model shown in Figure 7.

Parameters	F1	F2
Fv′/Fm′	1.152	-0.099
qP	2.491	-0.130
Φexc	1.105	-0.056
Φ_{NO}	-2.570	0.247
Φ_{NPQ}	-2.361	-0.225
Φ_{PSII}	0.081	-0.131
DIo/RC	-1.153	0.230
REo/RC	-1.266	0.074
RC/ABS	-1.097	-0.177
Wk	1.889	0.266
φPo	2.992	0.057
ψEo	-0.237	-0.107
φEo	-0.993	-0.085
Vj	-0.032	0.136
